# Stress-Strain Curves and Modified Material Constitutive Model for Ti-6Al-4V over the Wide Ranges of Strain Rate and Temperature

**DOI:** 10.3390/ma11060938

**Published:** 2018-06-02

**Authors:** Xin Hou, Zhanqiang Liu, Bing Wang, Woyun Lv, Xiaoliang Liang, Yang Hua

**Affiliations:** 1 School of Mechanical Engineering, Shandong University, Jinan 250061, China; houxin@mail.sdu.edu.cn (X.H.); sduwangbing@sdu.edu.cn (B.W.); sdulvwoyun@gmail.com (W.L.); sduliangxiaoliang@gmail.com (X.L.); sduhuayang@gmail.com (Y.H.); 2 Key Laboratory of High Efficiency and Clean Mechanical Manufacture of MOE/Key National Demonstration Center for Experimental Mechanical Engineering Education, Jinan 250061, China

**Keywords:** dynamic deformation, flow stress, constitutive model, Ti-6Al-4V

## Abstract

The mechanical properties of Ti-6Al-4V alloy are sensitive to strain rate and temperature load. The finite element simulation results of high-speed machining Ti-6Al-4V alloy depend on the accurate description of dynamic deformation. However, it is hard to describe the flow stress behavior in current constitutive models in a complex high-speed machining process for Ti-6Al-4V alloy. In this paper, the stress-strain curves of Ti-6Al-4V alloy under the wide ranges of strain rate and temperature are obtained by high-velocity uniaxial impact tests. The apparent coupling between temperature and strain is observed, which proves that the temperature is dependent on a hardening effect for Ti-6Al-4V alloy. A function describing the coupling between temperature and strain is then introduced into the modification for the original Johnson-Cook (JC) constitutive model. The maximum deviation between the predicted data from using the proposed modified JC constitutive model and experimental data is reduced from 10.43% to 4.19%. It can be concluded that the modified JC constitutive model is more suitable to describe the temperature-dependent hardening effect, which provides strong support for accurate finite element simulation of high-speed machining Ti-6Al-4V alloy.

## 1. Introduction

Titanium alloy has been widely employed in aerospace, energy and chemical industries. Ti-6Al-4V is the most commonly used titanium alloy due to its low density, high strength, and strong corrosion resistance [1]. However, a variety of factors affect the mechanical properties of Ti-6Al-4V alloy such as the initial microstructure, heat treatment, and chemical impurities [2]. Due to the low thermal conductivity and low modulus of elasticity, high-speed machining Ti-6Al-4V alloy is faced with some challenges [3]. The vibration caused by the formation of serrated chip and heat concentrating on the tool rake face during the machining process results in rapid tool wear and poor surface integrity [4]. The selections of tool geometry and optimum cutting parameters are of great significance to improve the processing efficiency and surface integrity [5].

The finite element simulation has been applied to the high-speed machining to optimize the cutting conditions. Due to the multi factors that affect the machining precision and surface integrity, the finite element simulation of machining is a very complicated process. In addition, it is prerequisite to establish the models of flow stress, strain, strain rate and temperature for workpiece materials. In previous studies, a large number of modeling works have been carried out, which mainly focuses on the effect of chip segmentation on tool chatter [6] and the machining parameters on surface integrity [7]. The accurate simulation to solve above problems by finite element simulation depends on the accuracy of the constitutive model of the workpiece material to be machined.

The constitutive model is used to describe the effects of strain, strain rate, and temperature on the dynamic behavior of the machined material. The computer program can simulate the dynamic deformation of the material under a specific loading condition through the constitutive model. However, it is difficult to accurately describe the deformation behavior of the material under different loading conditions in one constitutive model. A variety of constitutive models have been established, which can be roughly divided into two different types as empirical ones and physical ones. Empirical constitutive models are usually based on the uniaxial stress response of materials to obtain material flow stress, such as Johnson–Cook (JC) model [8], Khan–Huang–Liang (KHL) model [9], etc. Physical constitutive models are based on the changes in the physical state of the material, such as Baumann-Chiesa-Johnson (BCJ) model [10], Mechanical Threshold Stress (MTS) model [11], etc.

Another challenge is to obtain accurate constitutive model parameters of Ti-6Al-4V alloy. In the high-speed machining process, Ti-6Al-4V is faced with extreme deformation conditions (e.g., elevated temperature, high strain, and high strain rate), which are hard to be achieve by ordinary test methods for measuring material properties. Data used for the machining model at elevated temperature are obtained by preheating specimens, which is different from the rate of temperature change during machining. The material temperature rises more rapidly in machining than the temperature rise from preheating. The metallurgical processes at different heating rates are different, which will affect the material mechanical behaviors. However, the work of Hokka et al. proved that the deviation of different heating rates may not be significant [12].

Although researchers have established a series of constitutive models and modified models for Ti-6Al-4V alloy [13,14,15], the difficulties still remain with accurately describing the dynamic deformation behavior in high-speed machining Ti-6Al-4V alloy. In this paper, the stress-strain curves of Ti-6Al-4V alloy under loadings of different levels of strain rate and temperature are investigated by the quasi-static compression test and the dynamic compression test. Based on the experimental data, a new modified constitutive model is established. The modified constitutive model was proven to be more accurate in expressing the dynamic deformation behavior of Ti-6Al-4V alloy under high strain rate and elevated temperature.

## 2. Materials and Methods

### 2.1. Materials

Ti-6Al-4V is an alloy that includes 6 weight percent Al and 4 weight percent V. Due to different structures, the two phases of Ti-6Al-4V, known as α and β phase, have different properties [16,17]. In general, the microstructure of Ti-6Al-4V alloy is affected by the thermal coupling. The phase transition temperature of Ti-6Al-4V alloy is 1010 °C [18]. The main chemical compositions of Ti-6Al-4V alloy in this research are shown in Table 1.

The specimens of Ti-6Al-4V were prepared by wire electro-discharge machining. To control the deformation strain rate of the specimens during the test within a wide range of conditions, two different sizes of cylindrical specimens were cut along the axial direction of Ti-6Al-4V bar. One specimen was 3 mm in diameter and 3 mm in height, the other was 2 mm in diameter and 2 mm in height. In the machining process, the bottom and top surfaces of the specimen were ground and polished to ensure the precision to minimize the friction between the specimen and the device.

### 2.2. Quasi-Static and Dynamic Impact tests

The key to research the dynamic deformation behavior of Ti-6Al-4V alloy is to measure the mechanical properties under dynamic loadings such as Yield Strength (YS), work hardening, and toughness. At high strain rate and elevated temperature conditions, several experimental methods such as split Hopkinson pressure bar (SHPB) test (self-made device) [19], impact test (Instron, Boston, MA, USA.) [20], and drop hammer test (Yonekura, Osaka, Japan) [21] have been applied. The SHPB device is simple in structure. It is convenient in control and accurate in measurement. Meanwhile, the strain rate of specimen in the SHPB test can reach greater than 10^3^ s^−1^. Therefore, the SHPB device was used to test the dynamic deformation of Ti-6Al-4V alloy under the conditions of a large range of strain rate and temperature.

**Figure 1:**
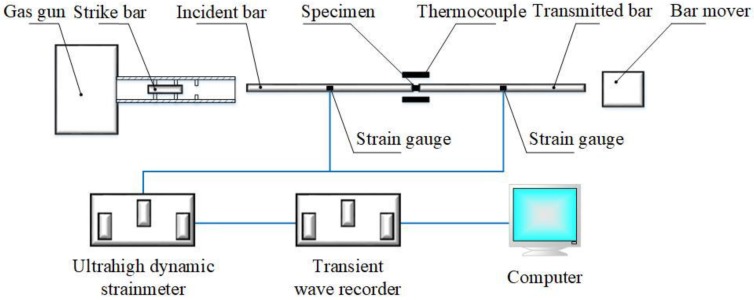
Schematic diagram of split Hopkinson pressure bar.

The SHPB device used in this research consisted of the strike bar, the incident bar, the transmitted bar, the bar mover, supporting equipment, and data acquisition system as shown in Figure 1. The specimen was placed between the incident bar and the transmitted bar. The strike bar drove the incident bar at a certain impact speed and resulted in a rectangular stress pulse. Then the stress pulse was forwarded in the incident bar. When the stress pulse reached the contact surface between the incident bar and the specimen, a portion of the stress pulse was reflected along the incident bar, and the other part continued through the specimen to the transmitted bar. The stress wave information of the incident bar and the transmitted bar was collected by the strain gauge pasted on the bar. The relationship among the stress, displacement, and time of the contact surface between the specimen and bar was solved.

In order to make the strain rate closer to the state in high-speed machining, the diameter of the incident bar and the transmitted bar were reduced to 5 mm. The maximum strain rate increased from 10^3^ s^−1^ to 10^4^ s^−1^. In this work, a series of tests were carried out to obtain the dynamic deformation over the wide ranges of strain rate and temperature. The quasi-static compression tests were carried out on a WDW-10 Test Machine with the maximum load 5 kN.

The SHPB tests were performed at strain rate ranging from 4000 s^−1^ to 12,000 s^−1^ and temperature ranging from 25 °C to 600 °C. In the high-temperature SHPB tests, the temperature of the specimen was controlled by the resistance wire heating furnace. Through the thermocouple connected closed-loop controller, the furnace temperature error range was maintained at ±5 °C.

The strain gauges on the surface of the incident bar and the transmitted bar were used to measure the change of the stress pulse. After amplification, two groups of signal images were obtained on the data acquisition system. Then the signal data were processed by using the theory of one dimensional stress wave.

The average engineering stress, strain, and strain rate of the specimen were specified as Equation (1) to Equation (3), respectively.

(1) $$\sigma \left(t\right)=\frac{A_{b}E\varepsilon_{t}\left(t\right)}{A_{s}}$$

(2) $$\varepsilon \left(t\right)=\frac{2C_{0}}{L_{s}}\int_{0}^{t}\varepsilon_{r}\left(t\right)dt$$

(3) $$\dot{\varepsilon }=\frac{2C_{0}\varepsilon_{r}\left(t\right)}{L_{s}}$$

where *A_b_* is the cross-sectional area of the bar, *E* is Young’s modulus of the bar. *ε_r_* (*t*) and *ε_t_* (*t*) are expressed as the function of the reflection strain and the transmission strain on time *t*. *C_0_* is the wave velocity. *L_s_* is the length of the specimen.

The true stress, the true strain, and the true strain rate for deformation of specimens can be determined with Equations (4)–(6), respectively.

(4) $$\sigma_{T}=\sigma \left(1+\varepsilon \right)$$

(5) $$\varepsilon_{T}=\ln\left(1+\varepsilon \right)$$

(6) $${\dot{\varepsilon }}_{T}=\frac{d\varepsilon_{T}}{dt}$$

## 3. Results and Discussion

### 3.1. Dynamic Deformation Behavior

**Figure 2:**
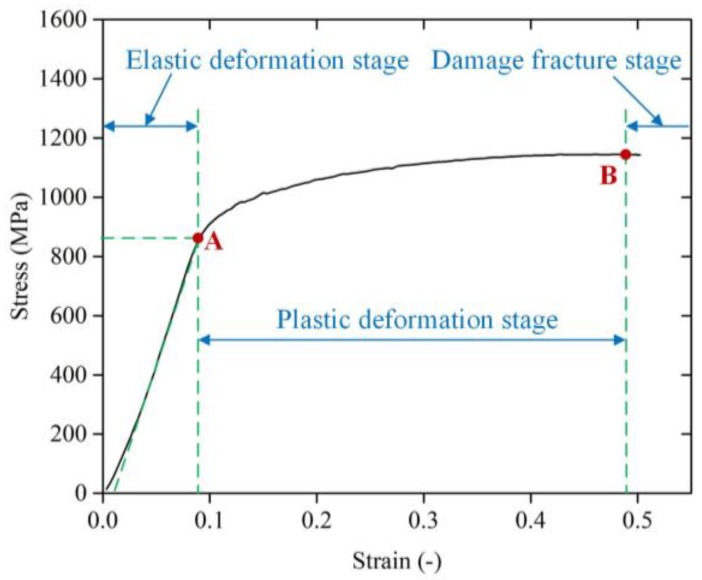
Stress-strain curve of Ti-6Al-4V alloy in the quasi-static test.

Figure 2 shows the stress-strain curve during the deformation of Ti-6Al-4V alloy in the quasi-static test. The curve OAB shows that Ti-6Al-4V alloy in the static compression process did not appear to obviously yield phenomenon. Thus, the stress at 0.2% plastic deformation is produced as the YS of the material, denoted by *σ*_0.2_, which is position A in Figure 2. For Ti-6Al-4V alloy used in the quasi-static test, *σ*_0.2_ is 920 MPa. The strain rate during the quasi-static compression test is 0.0017 s^−1^.

The strain hardening rate *Q* can be described as the rate of change of stress versus strain. The strain hardening rate can be approximated by Equation (7).

(7) $$Q_{i}=\frac{\partial \sigma }{\partial \varepsilon }=\frac{\sigma_{i}-\sigma_{i-1}}{\varepsilon_{i}-\varepsilon_{i-1}}$$

**Figure 3:**
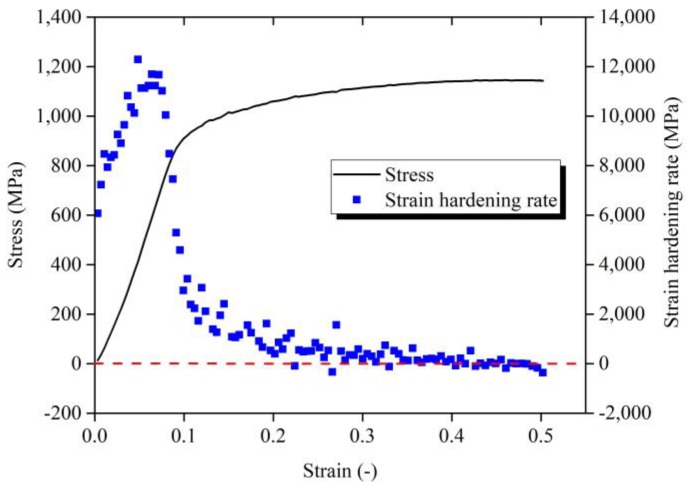
Strain hardening rate of Ti-6Al-4V alloy in the quasi-static test.

With using Equation (7), the strain hardening rate-strain behavior of Ti-6Al-4V alloy in quasi-static compression test can be obtained as shown in Figure 3. It can be seen from the strain hardening rate-strain behavior that the strain hardening rate of Ti-6Al-4V alloy shows a brief increase trend followed by a gradual decrease with the increase of strain. It means that the hardening trend of titanium alloy Ti-6Al-4V surges first and then decreases. During the elastic deformation stage, the strain hardening rate drops to zero. The hardening tendency of Ti-6Al-4V alloy is gradually reduced. After the plastic deformation stage, the strain hardening rate of Ti-6Al-4V alloy falls to a negative value, that is, Ti-6Al-4V alloy appears to have softening behavior.

**Figure 4:**
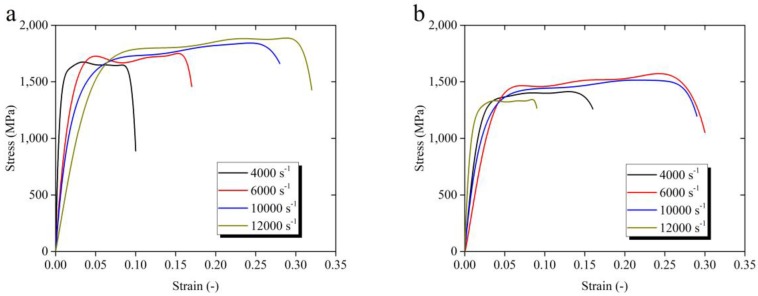

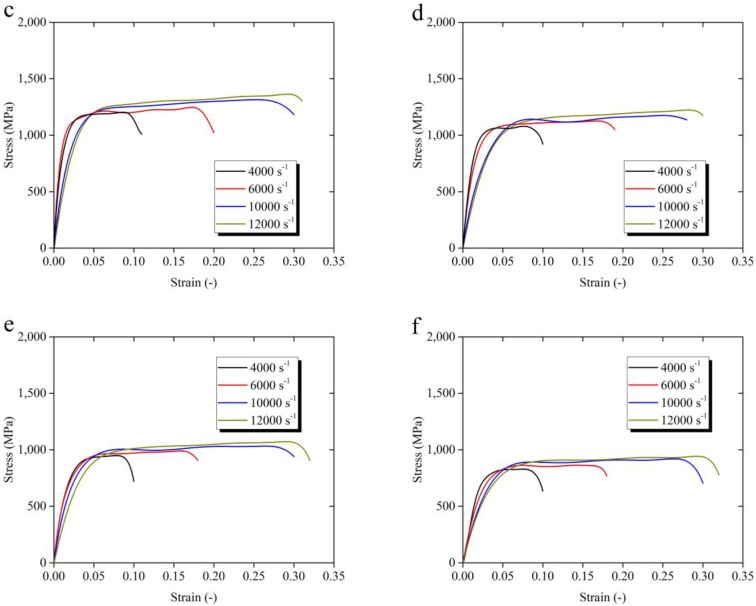
Stress-strain curves of Ti-6Al-4V alloy over the wide range of temperature of (**a**) 25 °C, (**b**) 100 °C, (**c**) 200 °C, (**d**) 300 °C, (**e**) 400 °C, and (**f**) 500 °C in SHPB test.

In Figure 4a–f, the stress-strain relations are presented over the wide range of temperature from 25 °C to 500 °C. Data under different levels of strain rate of 4000 s^−1^, 6000 s^−1^, 10,000 s^−1^, and 12,000 s^−1^ are collected. With the increase of strain rate from 4000 s^−1^ to 12,000 s^−1^ at room temperature (RT) shown in Figure 4a, the maximum value of the stress increases from 1649 MPa to 1872 MPa. At other temperatures in Figure 4b–f, the stress is observed to increase as the strain rate changes from 4000 s^−1^ to 12,000 s^−1^. However, as the strain rate increases, the rate of stress increase with the increasing strain does not change much.

**Figure 5:**
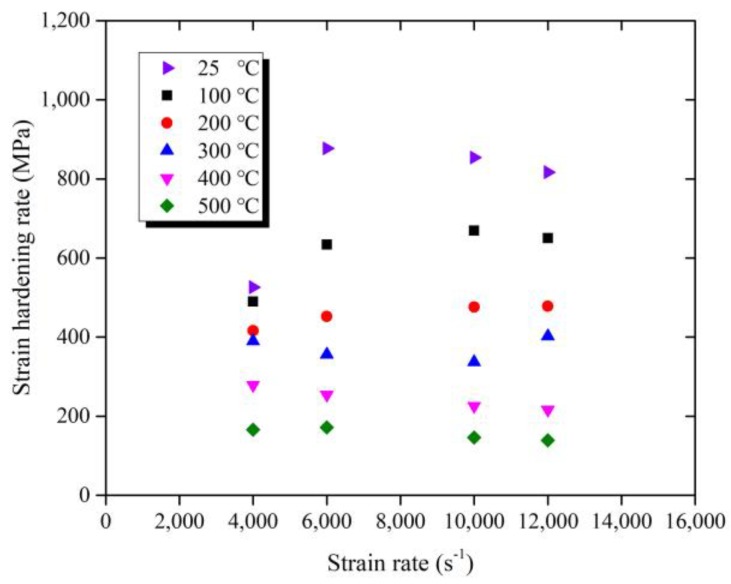
Strain hardening rate over the wide range of strain rate.

As shown in Figure 5, the strain-hardening rate over the wide range of strain rate in the process of plastic deformation was calculated to better describe the rate of stress variation with the rising strain, At the same temperature, the strain hardening rate has no obvious regularity with the rising strain rate. With the increase of temperature, the strain hardening rate at different strain rates tend to be similar, which indicates that Ti-6Al-4V alloy has no apparent strain rate sensitivity.

**Figure 6:**
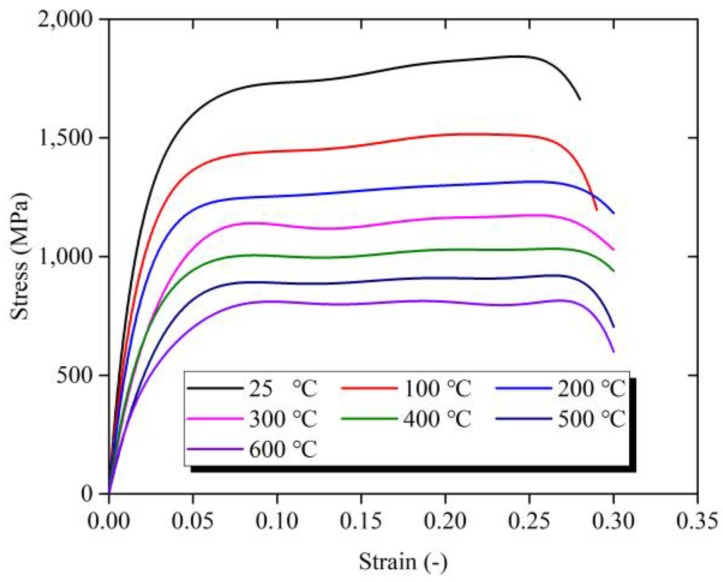
Stress-strain curves of Ti-6Al-4V alloy over the wide range of temperature from 25 °C to 600 °C in SHPB test.

**Figure 7:**
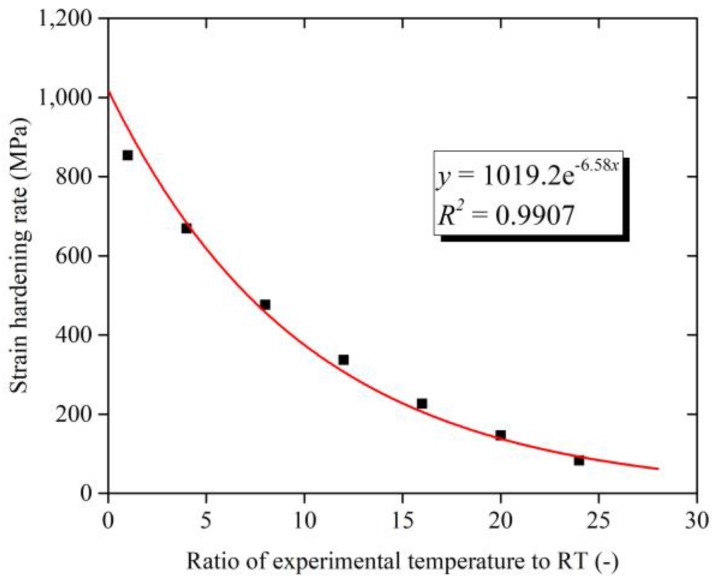
Strain hardening rate over the wide range of temperature.

The stress-strain curves over the wide range of temperature from 25 °C to 600 °C in SHPB test are shown in Figure 6. The data with the strain rate of 10,000 s^−1^ were selected. The maximum value of the stress decreases from 1842 MPa to 808 MPa with the rising temperature from 25 °C to 600 °C. With the increase of temperature, the rate of stress increase with the strain obviously drops. Figure 7 shows the relation between strain-hardening rate and temperature at the strain rate of 10,000 s^−1^. The x-coordinate represents the ratio of the experimental temperature *T* to the RT *T_r_*. The red line is a fitting curve according to the changes of strain hardening rate with temperature. The strain-hardening rate decreases with the increase of temperature, and the rate of strain-hardening rate decrease gradually slows down. The changing trend of strain-hardening rate with strain-increasing conforms to the law of power function, indicating that Ti-6Al-4V alloy has apparent temperature sensitivity.

### 3.2. Modified Constitutive Model

Compared to other constitutive models, the Johnson–Cook (JC) constitutive model has simple mathematical form, less material parameters, and good compatibility with finite element software. In addition, the JC constitutive model is suitable for describing the dynamic mechanical properties of nonferrous metals and difficult-to-machine materials [8]. That is why many researchers used the JC constitutive model to describe the flow stress behavior of Ti-6Al-4V alloy in high-speed machining process [22,23,24].

The JC constitutive model includes several parameters, including static YS, hardening modulus, strain hardening exponent, strain rate sensitivity coefficient, and temperature sensitivity coefficient. As an empirical model, the JC constitutive model describes the functional relationships of strain effects, strain rate effects and temperature effects in the form of product as shown in Equation (8).

(8) $$\sigma \left(\varepsilon ,\dot{\varepsilon },T\right)=\left(A+B\cdot \varepsilon^{n}\right)\left(1+C\ln\frac{\dot{\varepsilon }}{{\dot{\varepsilon }}_{ref}}\right)\left[1-{\left(\frac{T-T_{r}}{T_{m}-T_{r}}\right)}^{m}\right]$$

where *A* is the static YS. *B* is hardening modulus. *C* is strain rate sensitivity coefficient. *m* is thermal sensitivity coefficient. *n* is strain-hardening exponent. ${\dot{\varepsilon }}_{ref}$ is the reference strain rate. *T_r_* is RT, *T_m_* is the melting temperature.

The JC constitutive model can be divided into the product of the three terms. The term $\left(A+B\cdot \varepsilon^{n}\right)$ in Equation (8) represents the relationship between the stress and strain. The values of *A*, *B*, and *n* can be determined by stress-strain curves obtained under the RT and quasi-static loading conditions. The terms $\left(1+C\ln\frac{\dot{\varepsilon }}{{\dot{\varepsilon }}_{ref}}\right)$ and $\left[1-{\left(\frac{T-T_{r}}{T_{m}-T_{r}}\right)}^{m}\right]$ in Equation (8) represent the relationships between the stress and strain rate as well as temperature, respectively. The values of *C* and *m* can be obtained with the tests obtained under different levels of strain rate and temperature.

The JC constitutive model parameters for Ti-6Al-4V alloy in this study are calculated and obtained in Table 2 by fitting the three expressions.

Compared to the physical constitutive model, the empirical constitutive model lacks the principle of microstructure. In the JC constitutive model, the effect of dislocation density on flow stress is replaced by the effect of plastic strain. The dislocation density changes with the different microstructure stages, but the plastic strain cannot reflect the change of microstructure. On the other hand, JC constitutive model describes strain hardening as an increasing function without considering strain softening. It has been shown that the thermal softening has an important influence on the formation of the strain localization and adiabatic shear bands [25]. Hence, the thermodynamic term $\left[1-{\left(\frac{T-T_{r}}{T_{m}-T_{r}}\right)}^{m}\right]$ in Equation (8) also needs change with the plastic strain. The strain hardening rate *Q* can be described as Equation (9).

(9) $$Q=f(T^{*})$$

where T* is given by T* = *T*/*T_r_*. According to the fitting of the relation of strain hardening rate and temperature in Figure 7, it is found that Equation (10) can be described as a form of power function.

(10) $$Q=B(1+m_{1}\ln\frac{T}{T_{r}})$$

where *B* is the hardening modulus in RT, *m*_1_ is thermal sensitivity coefficient with the increasing strain. The modified JC constitutive model is then proposed and can be described as Equation (11).

(11) $$\sigma \left(\varepsilon ,\dot{\varepsilon },T\right)=\left[A+B\left(1+m_{1}\ln\frac{T}{T_{r}}\right)\varepsilon^{n}\right]\left(1+C\ln\frac{\dot{\varepsilon }}{{\dot{\varepsilon }}_{ref}}\right)\left[1-{\left(\frac{T-T_{r}}{T_{m}-T_{r}}\right)}^{m_{2}}\right]$$

The parameters in the modified JC constitutive model are calculated in Table 3.

**Figure 8:**
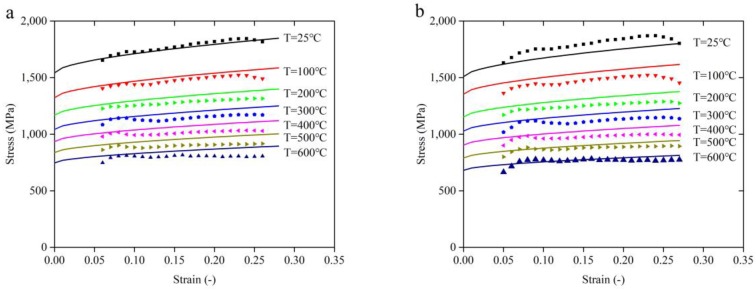

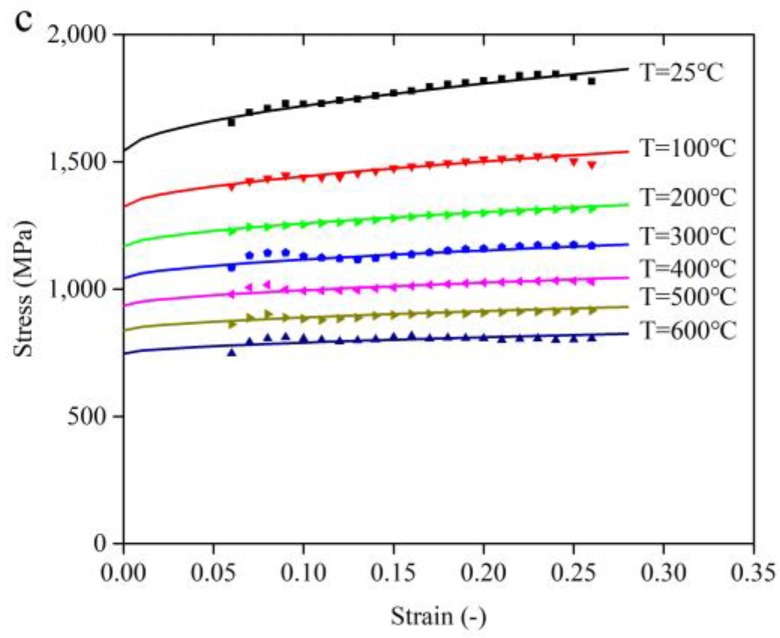
The comparison between the predicted and the measured stress over the wide range of temperature from 25 °C to 600 °C. (**a**) JC constitutive model, (**b**) Khan–Huang–Liang (KHL) constitutive model, (**c**) modified JC constitutive model.

The Khan–Huang–Liang (KHL) constitutive model is also widely used in the prediction of a material’s flow stress behavior in high-speed machining [23]. The comparison between the predicted stress obtained by JC constitutive model, modified JC constitutive model, and KHL constitutive model with the measured stress over a wide range of temperature from 25 °C to 600 °C is shown in Figure 8. When the temperature is below 200 °C, the predicted stress obtained both by JC constitutive model and the modified JC constitutive model agree well with the measured stress. As the temperature increases from 200 °C to 600 °C, the growth rate of predicted stress obtained by the JC constitutive model with the increasing strain is gradually higher than the measured stress. There is a clear error between the predicted and the measured stress, especially for the high temperature conditions. However, the KHL constitutive model shows the opposite effect. As the temperature increases from 200 °C to 600 °C, the prediction accuracy of the KHL constitutive model has been greatly improved. Correspondingly, the predicted stress obtained by the modified JC constitutive model agrees well with the measured stress.

**Figure 9:**
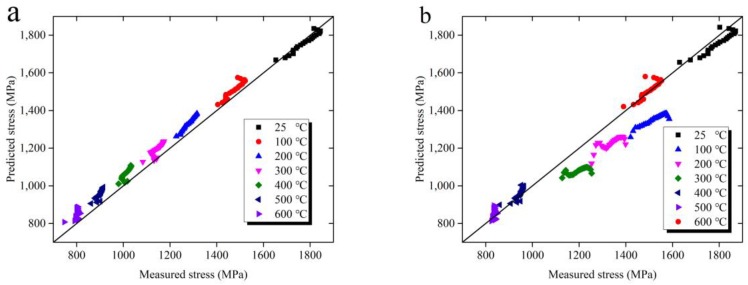

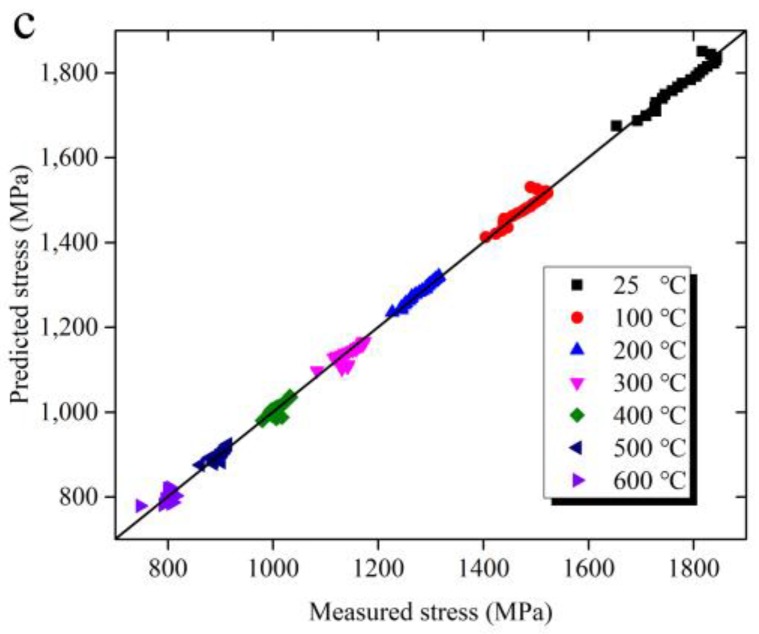
Correlation between the predicted and the measured stress over the wide range of temperature from 25 °C to 600 °C. (**a**) JC constitutive model, (**b**) KHL constitutive model, (**c**) modified JC constitutive model.

The correlation between the predicted and the measured stress at different temperatures from 25 °C to 600 °C is shown in Figure 9. With the temperature increases from 25 °C to 600 °C, the correlation degree becomes weak between the predicted stress obtained by JC constitutive model and the measured stress. The maximal deviation *δ* can be described as Equation (12).

(12) $$\delta =\max(\frac{\left|\sigma_{p}-\sigma_{m}\right|}{\sigma_{m}}\times 100\%)$$

where *σ_p_* is the predicted stress, *σ_m_* is the measured stress. The maximal deviations *δ* are 10.43% and 15.77% for the predicted stress obtained by JC constitutive model and KHL constitutive model with measured stress. However, the maximal deviation of the predicted stress obtained by modified JC constitutive model with measured stress is only 4.19%. Therefore, the modified JC constitutive model provides a better prediction accuracy for the flow stress behavior of Ti-6Al-4V alloy in high strain rate and elevated temperature.

## 4. Conclusions

In this work, the stress-strain curves of Ti-6Al-4V alloy under loading conditions over the strain rate range of 0.0016 s^−1^–12000 s^−1^ and the temperature range of 25–600 °C were investigated. The modified JC constitutive model has been proposed. The following conclusions can be drawn:

(1) The strain and temperature have obvious coupling effect on material dynamic behavior. The strain-hardening rate is dependent on the temperature. With the temperature increasing, the strain hardening rate drops and the strain hardening rate gradually decreases. The plastic deformation generated in high temperature leads to the decrease of hardening rate. Meanwhile, the coupling effect of strain rate and strain are not obvious;
(2) A modified JC constitutive model considering the coupling effect of temperature and strain is proposed. Experimental results show that the modified JC constitutive model provides a better prediction for flow stress behavior of Ti-6Al-4V alloy under loading conditions of high strain rate and high temperature.

## Figures and Tables

**Table 1:** Chemical compositions of Ti-6Al-4V.

Elements	Content (wt. %)
Fe	≤ 0.30
C	≤ 0.10
N	≤ 0.05
H	≤ 0.015
O	≤ 0.20
Al	5.50–6.80
V	3.50–4.50
Ti	rest

**Table 2:** The Johnson–Cook (JC) constitutive model parameters in this study.

Parameters	Value
*A* (MPa)	920
*B* (MPa)	380
*C*	0.042
*n*	0.578
*m*	0.633

**Table 3:** The modified JC constitutive model parameters in this study.

Parameters	Value
*A* (MPa)	920
*B* (MPa)	400
*C*	0.042
*n*	0.578
*m*_1_	0.158
*m*_2_	0.633
